# Understanding suicidal behavior and gender-specific pathways in an adolescent community sample: a structural model approach

**DOI:** 10.1007/s00787-025-02877-5

**Published:** 2025-10-30

**Authors:** Adriana García-Ramos, Sandra Doval, Rory O’Connor, Marco Innamorati, Andres Pemau, Alberto Sanchez, Wala Ayad-ahmed, Elizabeth Suárez-Soto, Jose Luis Ayuso-Mateos, Alejandro de la Torre-Luque

**Affiliations:** 1 Department of Psychiatry, School of Medicine, Universidad Autónoma de Madrid (UAM), Arzobispo Morcillo 4 Avenue, 28029 Madrid, Spain; 2 Centro de Investigación Biomédica en Red de Salud Mental, Instituto de Salud Carlos III, 28029 Madrid, Spain; 3 Department of Experimental Psychology, Cognitive Processes and Speech Therapy, Universidad Complutense de Madrid, Madrid, Spain; 4 Suicidal Behaviour Research Laboratory, School of Health & Wellbeing, College of Medical, Veterinary and Life Sciences, University of Glasgow, Glasgow, UK; 5 Università Europea Di Roma, Rome, Italy; 6 Department of Personality, Assessment, and Clinical Psychology, Complutense University of Madrid, Madrid, Spain; 7 Department of Clinical Psychology, and Psychobiology, Faculty of Psychology, University of Barcelona, Barcelona, Spain; 8 Department of Legal Medicine, Psychiatry and Pathology, School of Medicine, Universidad Complutense de Madrid (UCM), Madrid, Spain

**Keywords:** Adolescent suicide, Structural Equation Modeling, Gender Differences, Emotional abuse, Non-Suicidal Self Injury

## Abstract

Suicide remains a leading cause of death among adolescents, highlighting the need for more effective risk models to aid in prevention strategies. This study examined motivational and volitional factors contributing to suicidal ideation and attempts among adolescents aged 12–16 in a community-based sample., We investigated the roles of emotional abuse, non-suicidal self-injury, impulsivity, and emotional symptoms in predicting suicidal ideation intensity and likelihood of suicide attempt. We also investigated whether these associations differed by sex assigned at birth. Data were collected from 1,526 adolescents (M = 13.8; SD = 1.27) in Madrid, Spain, and analyzed under a Multiple Indicators and Multiple Causes (MIMIC) structural equation model. This Structural Equation Model (SEM) showed good fit (CFI = .96, TLI = .97, RMSEA = .078, SRMR = .061). Emotional symptoms and emotional abuse were significant predictors of ideation, while non-suicidal self-injury and ideation intensity were strongly associated with suicide attempt probability. A multigroup analysis showed that gender assigned at birth did not significantly moderate the relationship between risk factors and suicidal outcomes. However, females were more impacted by emotional abuse and emotional symptoms. These findings highlight the importance of inclusive, comprehensive prevention strategies that address universal and gender-specific risk factors in adolescent suicide prevention. Longitudinal studies are recommended to further explore the temporal dynamics of these associations.

**Supplementary Information:**

The online version contains supplementary material available at 10.1007/s00787-025-02877-5.

## Introduction

Suicide is among the leading causes of death in adolescents and young people (World Health Organization [WHO], [1, 2]). The WHO has declared suicide to be a global health problem, emphasizing the need to create and implement prevention strategies to curb mortality trends in the upcoming years.

Recent studies emphasize the concerning prevalence of suicide risk among younger adolescents, with prevalences of almost 30% in school settings (Fonseca-Pedrero et al., [3]), who report suicidal thoughts and 4.2%−17% who have attempted suicide at least once in their lives [4–6]. Importantly, suicidal ideation has been identified as one of the strongest and most consistent predictors of suicide attempts in adolescence [5, 7], reinforcing the need to examine both outcomes jointly when assessing suicide risk.

Suicide is a complex, multifaceted phenomenon [8]. A particularly influential theoretical framework for understanding the development of suicidal behavior is the Integrated Motivational-Volitional (IMV) Model (O’Connor & Kirtley, [9]). This model distinguishes between motivational factors that contribute to the emergence of suicidal ideation and volitional factors that facilitate the transition to suicidal behavior. In the present study, we draw on this framework to guide the selection of variables, as well as their availability within the proyect dataset, ensuring that the model captures key motivational and volitional components described in prior research.

Several individual and contextual risk factors have been consistently linked to adolescent suicidality. Perfectionism in response to parental criticism [10, 11], difficulties in emotion regulation [2, 11, 60], and limited perceived support from parents or peers [12–15] have all been associated with higher suicidal ideation. Moreover, exposure to adverse experiences such as childhood emotional abuse further undermines trust and access to social support, consistently elevating risk [16]. Together, these findings underscore the pivotal role of family and social context in shaping adolescent suicide risk.

In addition to individual and family-related factors, recent work has emphasized the role of broader social determinants and the need to humanize care in suicide prevention [55, 61]. Although these aspects were beyond the scope of the present study, they provide an important context for interpreting adolescent suicidal behavior.

Finally, alongside risk factors, research has also highlighted the importance of protective factors in reducing vulnerability to suicidal behavior among adolescents. Higher levels of perceived social support, strong family cohesion, emotional regulation skills, and school connectedness have been consistently linked to lower risk of suicidal ideation and attempts [17–19].

In addition to these psychological and behavioral variables, sociodemographic characteristics such as gender and age may shape how these risk factors manifest or interact. For example, the well-documented gender paradox (more suicide attempts in women but more deaths in men) has also been described in adolescents [20]. A meta-analysis by Miranda-Mendizabal et al. [21] further demonstrated that suicide risk factors vary substantially by sex in adolescents. Females showed higher risk in association with depressive symptoms, and interpersonal problems, while males were more strongly affected by externalizing problems, parental separation, and access to lethal means.

Although the IMV model has gained empirical support in adult and clinical populations, its application to adolescents remains relatively limited. A small but growing body of research has begun to test the model’s components in adolescent samples, particularly within cross-sectional designs [22, 23]. Empirical evidence for volitional phase variables, such as acquired capability or self-injury exposure, is still scarce in adolescents. Moreover, existing studies are concentrated in specific cultural contexts (primarily Asia and Germany), limiting cross-cultural generalizability [24, 25].

Within this framework, we define motivational factors as those contributing to the emergence of suicidal ideation—such as emotional symptoms, emotional abuse, perfectionism, and perceived lack of social support. In contrast, volitional factors—including impulsivity and the presence of non-suicidal self-injury (NSSI)—are understood as facilitators of the transition from ideation to suicidal behavior.

Based on this approach, the current study aims to examine the structure and magnitude of these associations in a community-based sample of adolescents aged 12 to 16, with particular attention to potential differences by sex assigned at birth. We hypothesize that emotional symptoms, emotional abuse, low perceived social support, and perfectionism concerns (motivational factors) will be positively associated with the intensity of suicidal ideation. We also hypothesize that non-suicidal self-injury (NSSI) and impulsivity (volitional factors), as well as suicidal ideation intensity itself, will be positively associated with the likelihood of a suicide attempt. In line with previous research, we expect that suicidal ideation will significantly predict suicide attempts, reflecting the ideation-to-action transition described in existing models.

Finally, we explore whether sex assigned at birth moderates the strength of these associations. Given mixed findings in the literature, this analysis is considered exploratory, although prior studies suggest that certain predictors, such as emotional symptoms or abuse, may exert stronger effects in female adolescents.

**Fig. 1:**
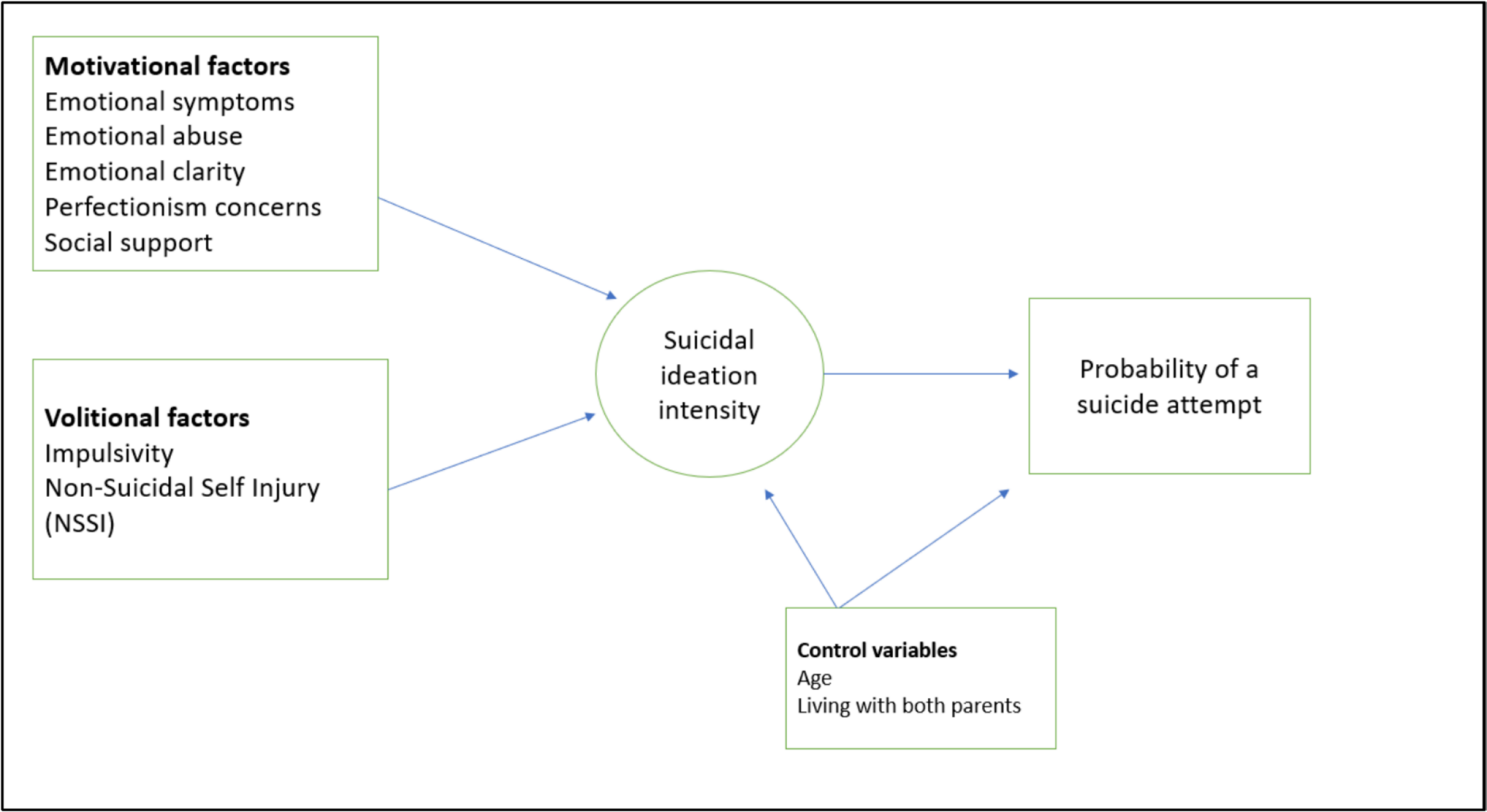
Conceptual framework of hypothesized relationships between motivational and volitional factors, suicidal ideation, and suicide attempt

A visual representation of these hypothesized relationships is presented in Fig. 1.

Despite the growing body of research on suicidal behavior in adolescents, there is still limited empirical work that simultaneously integrates both motivational and volitional predictors within a structural framework in large, community-based samples. Most existing studies either focus on clinical populations, isolate specific predictors, or overlook the structural differentiation between ideation and attempt. Moreover, there remains inconsistency in how sex differences are conceptualized and tested within these models, which complicates the development of inclusive and generalizable prevention strategies. By addressing these gaps, the present study advances the field by providing a theoretically guided, empirically tested model of suicidal risk in adolescents, clarifying the distinct roles of key psychological, behavioral, and contextual factors, and examining their relevance across sexes. These insights are intended to inform more nuanced and comprehensive prevention programs, particularly in early adolescence.

## Methods

### Design and procedure

This study analysed data from, “The EPISAM-School project”. This project aims to detect suicidal ideation in adolescents aged 12 to 16 in school settings, as well as associated risk factors. Schools in the Community of Madrid region (Spain) were randomly selected based on the census of schools offering secondary education. Subsequently, the school boards were contacted via an email letter that provided information about the study's characteristics, its voluntary and free nature, and the ethical specifications. The research project was designed in accordance with the ethical principles outlined in the Declaration of Helsinki and current legislation related to the protection of personal data [56] and received favorable approval from the corresponding ethics committee (REF 22/633-E).

The questionnaires were hosted online on the National Platform for the Study and Prevention of Suicide. The data collection took place between January and May 2023. All participants completed the questionnaires using an anonymized code provided during the session.

### Participants

The study included a sample of 1,526 adolescents (range 12–16 years; *M[mean]* = 13.8; *SD[standard deviation]* = 1.27) recruited from 10 schools in the previously mentioned area. The schools were invited to participate upon school board and parent association acceptance. The schools recruited consisted of four public and six semi-private schools. The initial sample comprised 1,647 adolescents evaluated over a period of five months, and data from 1,526 (92.5%) of them were usable due to complete information and unfilled questionnaires from the remaining participants. Because incomplete cases did not provide sufficient sociodemographic or clinical data, it was not possible to statistically compare them with included participants.

After obtaining informed consent from both the minors and their legal guardians, the assessment protocols were conducted in the classrooms of the participating schools. The participants completed the screening questionnaires online on individual computers, ensuring confidentiality and anonymity during the process. Information about support and mental health resources was available upon request, allowing participants to access assistance if needed.

The inclusion criteria were: (1) attending an educational center in the municipal districts of the Community of Madrid (Spain), (2) proficiency in Spanish, and (3) being between 12 and 16 years old. The exclusion criteria included: (1) having a diagnosed autism spectrum disorder or neurodevelopmental disorder and (2) having a severe sensory deficit that impeded reading or hearing. No additional statistical outliers were removed from the data, beyond the exclusion of cases with incomplete or missing responses.

### Measures

The present study was conducted within the framework of a large-scale school-based initiative. As such, the selection of variables for the current model was based on the measures available in the broader dataset. These variables were mapped onto motivational and volitional domains of the IMV model, prioritizing constructs with both theoretical relevance and empirical support in adolescent populations. Specific variables relevant to the study were selected from the original data set, which included measures of:

- *Paykel Suicide Scale (PSS).* A five-item self-report scale used to measure the severity of suicidal ideation in adolescents, addressing various aspects of both suicidal thoughts and behaviors [26]. For this study, we only used the total score for ideation items, ranging from 0 to 4. The response for each item is binary, “Yes” or “No”. The Paykel Suicide Scale demonstrated adequate reliability levels in Spanish validation studies (α = 0.93, ω = 0.82) [27] and in the current sample (α = 0.77).
- *Suicidal Behaviors Questionnaire-Revised (SBQ-R).* The SBQ-R is a brieffour-item tool designed to assess the risk of suicide [28, 29] with a good reliability (α = 0.88). In this study we used item 4, which refers to the likelihood of suicide. The Spanish validation carried out by [(57] supported good reliability of the measure (α = 0.81) and for this sample, the internal consistency was acceptable (α = 0.82).
- *Inventory of Statements About Self-Injury (ISAS)*. A 39-item questionnaire divided into two sections and developed by Klonski & Glenn (58) with an alpha range from 0.80 to 0.88. The Spanish version was validated by Pérez et al. [30], who reported an alpha ranging from 0.87 to 0.89. The item regarding the presence or absence of self-injury behavior was used for this study.
- *Strengths and Difficulties Questionnaire (SDQ).* A 25-item self-report tool used to identify emotional and behavioral problems in children and adolescents aged three to 16 years [31], validated in Spain by Ortuño-Sierra et al. [32]. For this study, only the emotional symptoms subscale was used (α = 0.72 and ω = 0.71). Responses are given in a 3-point Likert format. For this sample, the internal consistency was (ω = 0.71).
- *Multidimensional Scale of Perceived Social Support (MSPSS)*. A 12-item scale designed to identify areas where a person may feel a lack of support and to assess the relationship between perceived social support and various mental health and well-being outcomes (Zimet et al., [33]) (α = 0.85–0.91). It was adapted in a Spanish sample by López Ramos et al. [34] with an α = 0.92–0.94. Responses are given in a seven-point Likert-type format. It shows strong psychometric properties with internal consistency ranging from (α = 0.85 to 0.95) (Gavino, et al. [35]). For this sample, Cronbach’s α was 0.94.
- *Difficulties in Emotion Regulation Scale (DERS).* A 36-item scale designed to measure difficulties a person may have in regulating their emotions [36]. Responses are given in a five-point Likert-type format. It was validated in a Spanish sample by Hervás & Jódar [37]. For this study, only the subscale of lack of Emotional clarity and Impulsivity issues were used. Cronbach’s alphas were 0.84 and 0.86 in the original validation study [36], 0.78 and 0.80 in the Spanish validation study (Hervas et al., 59), and (α = 0.79 and 0.86) for this sample.
- *Frost Multidimensional Perfectionism Scale (FMPS)*. This scale consists of 35 items with a five-point Likert-type response format. It aims to evaluate perfectionism from a multidimensional perspective, recognizing that perfectionism can manifest in various ways and have different origins and consequences [38]. We used only the subscale “Concern over mistakes” for this study, based on the Spanish validation carried out by Gavino et al. [35]. The overall internal consistency and that of its subscales is strong (α = 0.87 to 0.93) [35], with values for this sample being (α = 0.91).
- *Childhood Trauma Questionnaire (CTQ-SF)*. A 28-item questionnaire with a five-point Likert response format designed to assess the degree and type of trauma experienced during childhood and adolescence. It was developed by Bernstein et al. [39] and validated in the Spanish sample by García-Fernández et al. ([40]). Only the emotional abuse scale was used (α = 0.83–0.94) [41]. The internal consistency of the scale was found to be strong (α = 0.84).

Altogether, these measures were chosen to represent motivational (emotional abuse, emotional symptoms, perfectionism, social support) and volitional (NSSI, impulsivity) domains within the IMV framework, allowing us to test both types of predictors simultaneously.. Additional information on socioeconomic status or cognitive ability (e.g., IQ) was not collected.

The assessment was conducted during regular school hours in classroom settings and required approximately one hour to complete.

### Data analysis

**Fig. 2:**
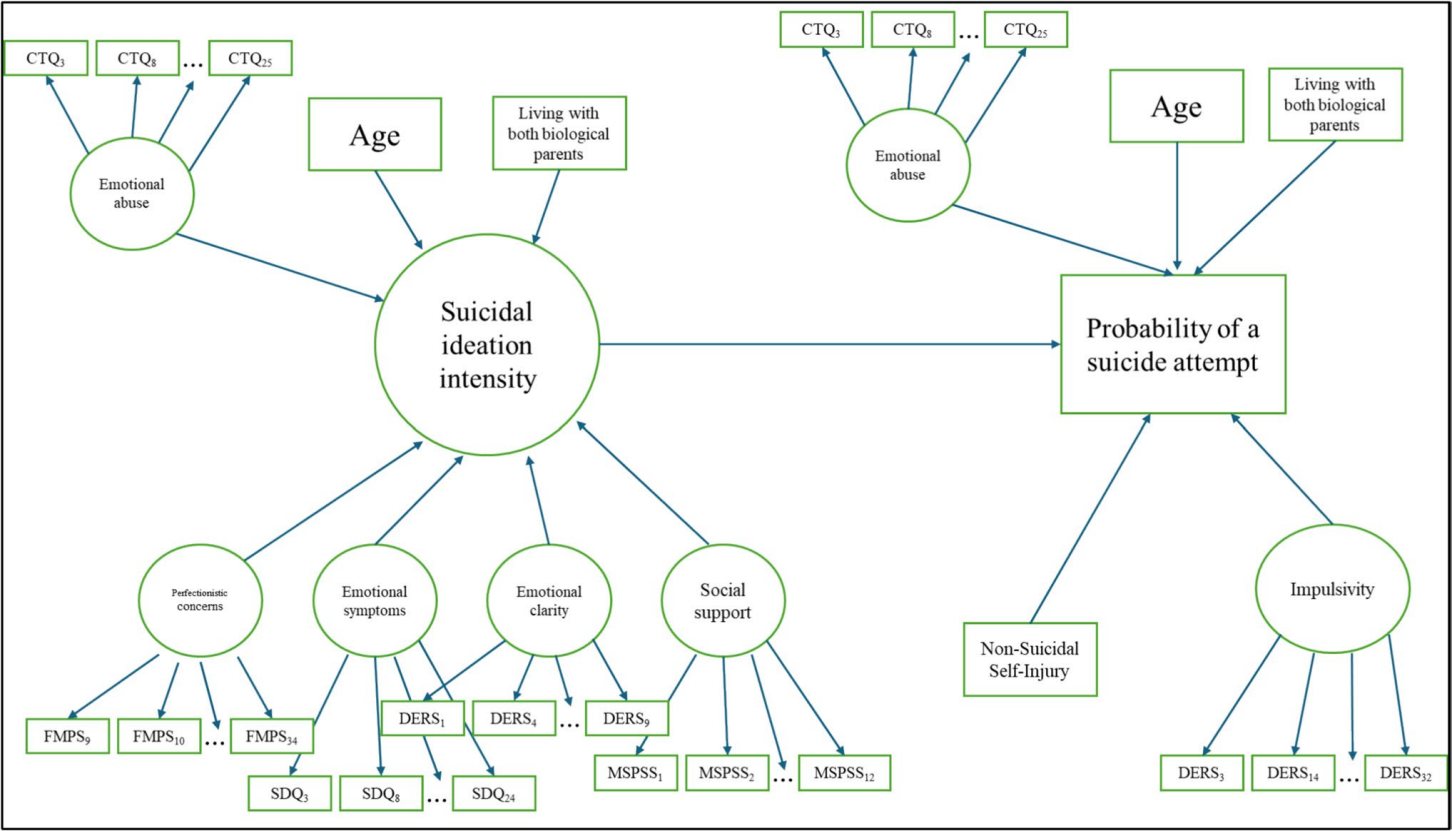
Structural suicidal risk model in early adolescents from the community. *Note.* Squares depict observed variables and circles depict latent variables. Straight arrows represent regression paths

Multiple indicators and multiple causes (MIMIC) modeling was used to explain the main forms of our suicidal risk model: suicidal ideation intensity (from the PSS) and the probability of engaging in a suicide attempt (SBQ item), it was employed to estimate direct effects of both observed and latent predictors on suicidal ideation intensity and suicide attempt likelihood. Additionally, a multigroup MIMIC approach was used to assess potential moderation effects by sex assigned at birth through a stepwise invariance testing procedure..Contributing (latent) factors for suicidal ideation considered were: perfectionistic concern over mistakes (FMPS), emotional symptoms (SDQ), lack of emotional clarity (DERS), and social support (MSPSS). Volitional factors hypothesized to be associated with probability of engaging in a suicide attempt (alongside with suicidal ideation intensity) were: the presence of non-suicidal self-injury (ISAS) and latent factor of impulsivity (DERS). Two sociodemographic factors were considered, hypothesized to be associated with both main forms of suicidal risk: age and not living with both biological parents. Finally, the exposure to emotional abuse (CTQ-SF) was also considered a motivational factor contributing to both the suicidal ideation severity and the probability of engaging in a suicide attempt. Figure 2 displays the path diagram of the structural model. All statistical analyses were conducted using RStudio (version 2023.12.1) employing the *lavaan* package for SEM modeling.

First, a MIMIC model was run to test the main effects of contributors on suicidal risk forms. To test for sex-related effects, multigroup MIMIC was conducted, under the measurement invariance tradition [42]. The biological sex at birth was used as a multigroup factor. Measurement invariance was based on a stepwise strategy, to compare the fit of increasingly restrictive models (i.e., being model parameters increasingly constrained to be equal between groups): the unconstrained model (configural invariance), constraining sex-specific solutions to fit on a same structural model,item loadings to be equal between groups, on the metric invariance model; the scalar invariance model adding an additional constraint on item intercepts; and regressor invariance, adding constraints on exogen factor loadings to endogenous factors (suicidal ideation intensity and the probability of engaging in a suicide attempt). MIMIC solution parameters were estimated using diagonally weighted least squares (DWLS) methods due to categorical exogenous and endogenous variables.

The following fit indexes were considered to assess goodness-of-fit of the MIMIC models: the χ^2^ statistic, the root mean square error of approximation index (RMSEA) with a 90% confidence interval, the comparative fit index (CFI), the Tucker-Lewis index (TLI), and the standardized root mean square residual (SRMR). Good fit of a model may be upheld by values of RMSEA < 0.08, CFI ≥ 0.95, TLI ≥ 0.95, and SRMR < 0.08 [43]. Multigroup effects were examined by comparing nested models (e.g., unconstrained vs. metric invariance model, metric invariance vs. scalar invariance model, etc.) using the incremental RMSEA (ΔRMSEA), the incremental SRMR (ΔSRMR), and incremental CFI (ΔCFI). A significant decrement in model fit measured by those incremental indexes reveals a lack of measurement invariance. More concretely, ΔCFI < −0.010 and ΔRMSEA > 0.015 or ΔSRMR > 0.030 would reflect significant differences between nested models [44, 45], pointing a significant influence of the multi-group variable. It is important to note that the χ^2^ statistic is highly sensitive to sample size and may yield statistically significant results in large samples, even when other indices indicate good model fit. For this reason, we relied primarily on alternative fit indices (CFI, TLI, RMSEA, SRMR) to evaluate model adequacy.

All the analyses were conducted using R Core Software 4.3.2 using the lavaan package [46].

## Results

Table 1 shows the distribution of sample participants considering all the sociodemographic and clinical features. The sample consisted of 1526 adolescents (54.3% female, 45.7% male). The majority (98.2%) lived with both parents and most participants (86.9%) were born in Spain. Regarding mental health indicators, 43.2% of participants reported at least one instance of suicidal ideation in the past (PSS), and 23.9% reported a moderate to high likelihood of attempting suicide in the future (SBQ-R item 4). Additionally, 34% reported having engaged in non-suicidal self-injury (NSSI), and 6.7% scored above the clinical cut-off (≥ 13) for emotional abuse on the CTQ-SF, suggesting significant exposure to this type of adverse childhood experience.

**Table 1:** Descriptive statistics

Variable	Frequency (%)	Mean (Standard Deviation)
Gender assigned at birth		
Female	828 (54.3)	
Male	698 (45.7)	
Living with both parents		
“Yes”	1499 (98.2)	
“No”	27(1.8)	
Nationality		
Born in Spain	1169 (86.9)	
Born outside of Spain	176 (13.1)	
Suicidal ideation (PSS)	659(43.2)	0.89 (1.26)
Self-Harm (ISAS)	519(34)	1.36 (.48)
Likelihood of suicide (SBQ)		.78 (1.22)
Emotional abuse (CTQ)	102(6.7)	7.1 (3.77)
Concern over mistakes (FMPS)		20.89 (8.91)
Lack of emotional clarity (DERS)		11.18 (4.46)
Impulsivity issues (DERS)		12.46 (5.68)
Perceived social support (MSPSS)		69.52 (14.88)
Emotional symptoms (SDQ)	1141(74.8)	3.57 (2.40)

Descriptive statistics collected through the sociodemographic scale. Mean and standard deviation of the questionnaires used in the screening process are included

**Fig. 3:**
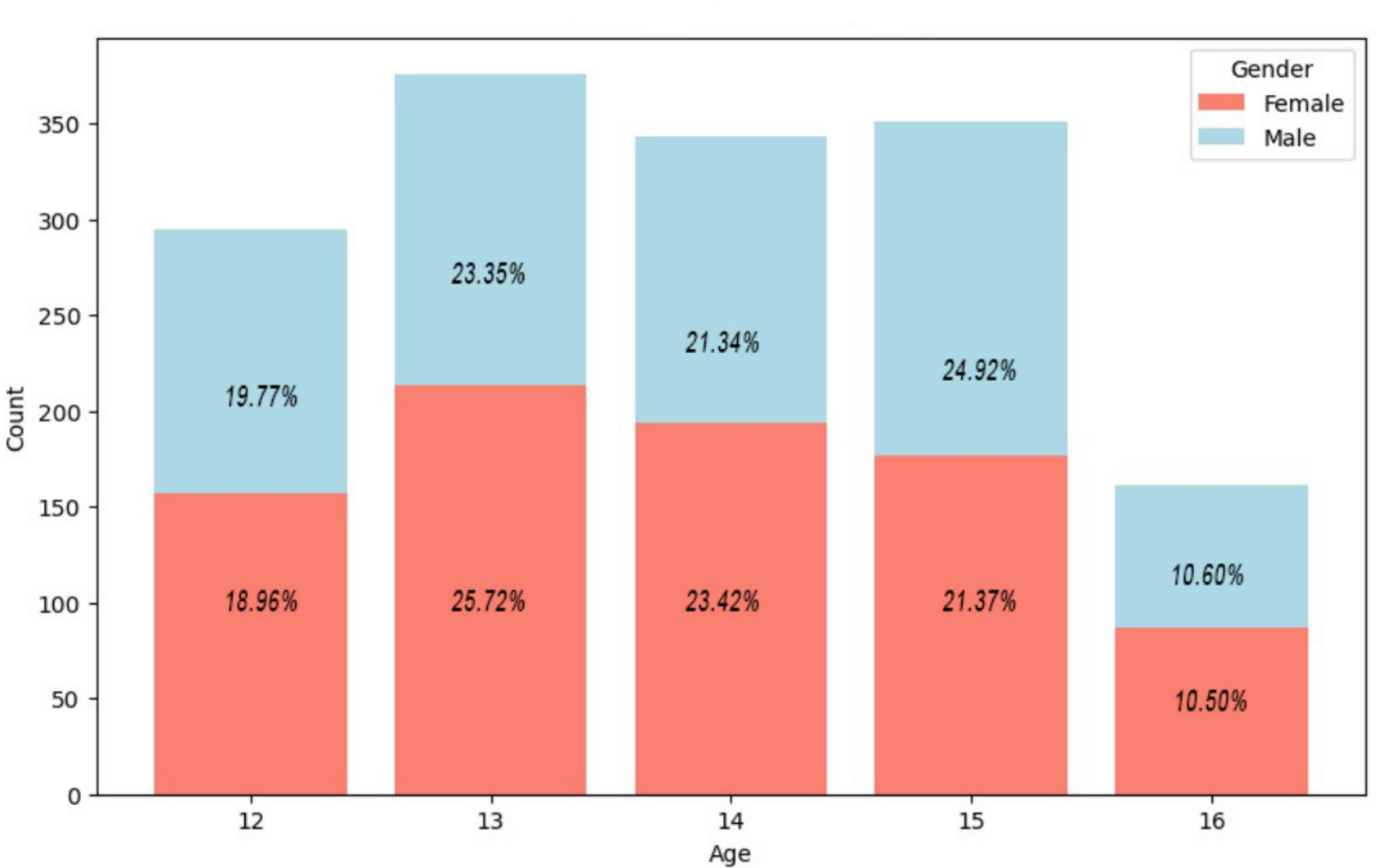
Distribution by age and gender assigned at birth

Distribution by age and gender assigned at birth can be seen in Fig. 3.

The MIMIC model depicting suicidal risk and covering the different suicide-related outcomes showed a good fit, given its sample size [47] with χ^2^ (1148) = 11,849.27, *p* < 0.01, CFI = 0.96, TLI = 0.97, RMSEA = 0.078 CI_90_ = [0.077, 0.079], SRMR = 0.061. Further model parameters can be found in Table S1, in Supplementary Material. We obtained adequate levels of explained variance for both endogenous variables, the suicidal ideation intensity (*R*^2^ = 0.61), and probability of engaging in a suicide attempt (*R*^2^ = 0.58). In terms of exogenous variables (contributors) of suicidal ideation intensity, four of them had loadings significantly different from zero: age (*β* = 0.10, *z* = 2.85, *p* < 0.01), living with both biological parents (*β* = 0.07, *z* = 2.16, *p* < 0.05), emotional symptoms (*β* = 0.62, *z* = 6.43, *p* < 0.01), and emotional abuse (*β* = 0.19, *z* = 3.35, *p* < 0.01).

In addition, higher ideation intensity was related to: older age, not living with both biological parents, higher levels of emotional symptoms, and higher emotional abuse. Regarding the probability of a suicide attempt, three exogenous variables were significant: suicidal ideation intensity (*β* = 0.51, *z* = 11.52, *p* < 0.01), non-suicidal self-injury (*β* = 0.45, *z* = 16.81, *p* < 0.01), and emotional abuse (*β* = 0.11, *z* = 2.64, *p* < 0.01). In this case, a higher ideation intensity, higher risk of non-suicidal self-injury, and more severe emotional abuse were associated with higher risk of a suicide attempt. MIMIC model parameters, Table S2 are displayed in the Supplementary material.

To test whether the network of relationships between endogenous and exogenous variables was different according to sex assigned at birth, multigroup MIMIC models were conducted under the measurement invariance framework. The comparison of increasingly constrained models indicated no significant decrement in model fit, supporting the assumption of measurement and structural invariance across groups (i.e., ΔCFI < −0.010 and ΔRMSEA > 0.015 or ΔSRMR > 0.030). Increasingly constrained model fit indexes are displayed in Table 2.

**Table 2:** MIMIC model fit and multigroup comparison

	MIMIC model fit indexes					Multigroup comparison		
	χ^2^ (df)	RMSEA (CI_90_)	CFI	TLI	SRMR	ΔCFI	ΔRMSEA	ΔSRMR
Configural	12,339.92 (2296)	.076 (.074,.077)	.968	.970	.066			
Metric invariance	12,718.52 (2348)	.076 (.075,.077)	.967	.969	.068	-.001	.001	.002
Scalar invariance	12,495.53 (2471)	.073 (.072,.074)	.968	.972	.066	.001	-.003	-.002
Regressor invariance	12,516.33 (2484)	.073 (.072,.074)	.968	.972	.066	.000	.000	.000

χ^2^ = χ^2^ test; df = degrees of freedom; RMSEA = root mean square error of approximation index (scores below.080 depict satisfactory model fit); CI = confidence interval at 90%; CFI = comparative fit index; TLI = Tucker-Lewis index. Scores of.95 or more indicate satisfactory model fitting, for TLI and CFI. SRMR = standardised root mean square residual (scores below.080 depict good fit)

An ΔCFI < -.010 and ΔRMSEA >.015 or ΔSRMR >.030 reflect significant differences between nested models (the simpler one would not be nested within the more complex one)

Thus, the overall model structure was similar for males and females. However, inspection of group-specific regression estimates (Table S3) revealed differences in the strength and significance of specific predictors. For instance, emotional symptoms and emotional abuse showed stronger associations with suicidal ideation among females, while emotional abuse was significantly associated with suicide attempt probability only among males. These differences should be interpreted with caution, as no formal statistical test of coefficient differences was conducted.

## Discussion

This study aimed to examine the main factors associated with suicidal ideation and suicide attempts among adolescents aged 12 to 16 in a school setting, using a structural equation modeling (SEM) approach. The model explored both motivational and volitional factors to understand their role in predicting suicidal outcomes. Additionally, the study sought to assess whether these relationships were moderated by biological sex or gender assigned at birth.

The proposed model showed a strong fit to the data, supporting its usefulness in explaining suicidal ideation and likelihood of attempt in this adolescent population. The fit of the model demonstrates the validity of the proposed risk model among adolescents ages 12 to 16, and the utility of motivational and volitional factors in predicting suicide ideation intensity and suicide attempt probability.

Several risk factors were found to be significant predictors of suicidal ideation intensity and the likelihood of a suicide attempt. Emotional symptoms had a particularly strong association with suicidal ideation. These findings align with existing literature that highlights the important role of emotional symptoms in suicidal behavior, especially in the youth population [48].

Emotional abuse was another factor correlated strongly with suicidal ideation, while non-suicidal self-injury and ideation intensity emerged as key predictors of the likelihood of future suicide attempts, aligning with previous research indicating the significant role of these risk factors [60]. Emotional abuse has also been associated with long-term emotional dysregulation, leading to heightened vulnerability to both ideation and attempts, as supported by studies on trauma and emotional neglect [49–51].

Our model supports a comprehensive understanding of how both motivational and volitional elements contribute to adolescent suicidal thoughts and behavior. It also highlights the complexity of factors associated with suicidal behavior among adolescents, providing a reliable framework for future research and intervention. Furthermore, findings underscore the importance of addressing both internalizing emotional difficulties and external stressors like trauma in adolescent suicide prevention strategies.

Additionally, the presence of non-suicidal self-injury emerged as a significant predictor of the likelihood of a suicide attempt, while a history of emotional abuse was identified as a predictor for both suicidal ideation and the likelihood of an attempt.

Sociodemographic factors, such as older age and not living with both biological parents, also played a role in increasing the risk of suicidal ideation, consistent with findings from previous research that links family structure and adolescent mental health outcomes [12]. These associations highlight the need for multifaceted interventions that address family dynamics.

In terms of sex differences, our multigroup MIMIC analysis suggested that while risk factors impact male and female adolescents similarly, certain factors influence females more intensely. These analyses revealed that biological sex does not play a significant role in moderating the relationships between the identified contributing factors and suicidal outcomes. This suggests that the relationships between emotional symptoms, impulsivity, NSSI, and suicidal outcomes are consistent across genders. However, the magnitude of some of them varies; females could experience a greater impact from emotional abuse and emotional symptoms, which is consistent with studies indicating that adolescent girls are more vulnerable to abuse situations [20] or more likely to experience relational forms of abuse, amplifying emotional dysregulation an ideation intensity [21] ​. This finding reinforces the importance for addressing several risk factors, such as emotional abuse, in prevention programs targeted at female minors.

In line with de IMV model, motivational factors such as emotional symptoms and emotional abuse were strongly associated with suicidal ideation, whereas volitional factors such as NSSI and impulsivity, together with ideation intensity, were predictive of suicide attempt probability. This differentiation between motivational and volitional domains provides empirical support for the applicability of the IMV model in community-based adolescent samples, an area where evidence is still scarce [24, 25].

Moreover, the fact that the moderating role of sex assigned at birth did not emerge as statistically significant, which resonates with the IMV’s proposition that contextual and individual vulnerabilities may operate similarly across groups, even if their magnitude differs. By integrating our findings into this theoretical framework, we contribute to clarifying the mechanisms by which risk factors escalate from ideation to attempts in adolescence, highlighting the need for early interventions targeting both motivational and volitional processes.

While our structural model did not reveal statistically significant gender differences, some predictors—such as emotional symptoms and emotional abuse—showed stronger associations in females. These trends, although not conclusive, may reflect developmental patterns that become more distinct over time. This supports the value of early, universal prevention strategies that address shared underlying mechanisms, while remaining attentive to the ways in which these mechanisms may differentially manifest across sexes, as suggested by previous research.

Likewise, although our findings diverge from studies reporting higher rates of suicidal ideation and NSSI in adolescent girls [52–54], they reinforce the need for inclusive and non-gendered approaches to intervention—without excluding the possibility that tailored strategies may still be beneficial in certain contexts.

These findings point to several concrete recommendations for school-based prevention programs. School-based programs should focus on universal prevention efforts and ensure access to mental health resources, particularly for students experiencing emotional distress, regardless of the specific underlying cause. The strong connection between emotional abuse and suicidal ideation suggests the need for trauma-informed approaches in school settings. This could include training school staff to recognize signs of emotional abuse and establishing clear protocols for intervention when such signs are detected. The similar pattern of risk factors across genders supports developing universal prevention strategies, while still maintaining sensitivity to how these factors might manifest differently in male and female students. In addition, prevention strategies should also seek to strengthen protective resources such as social support, family cohesion, and school belonging, which have been shown to reduce suicide risk in adolescents [18, 19].

To summarize, this study provides novel insights by applying a theoretically grounded structural model to a large, community-based sample of adolescents, distinguishing between motivational and volitional processes in the emergence of suicidal thoughts and behaviors. By demonstrating that these pathways are largely consistent across sexes—while also identifying specific vulnerabilities in girls—our findings contribute to refining current theoretical models of adolescent suicide risk. Importantly, the study addresses a gap in the literature by modeling both ideation and attempt likelihood within a unified framework. This research provides evidence that can inform interventions, emphasizing the need for coordinated, interdisciplinary approaches to promote adolescent mental health and reduce suicide risk.

## Limitations and future research

While the present study provides important evidence for understanding suicidal thoughts and behavior in the adolescent population, it has several limitations that should be considered when interpreting the findings. First, the cross-sectional nature of the design limits the ability to establish causal relationships between the factors analyzed and suicidal behavior. The continuation of data collection in the EPISAM-School Project has been planned as a longitudinal study, so future studies will be able to better understand the temporal dynamics and potential causal pathways more accurately.

Secondly, the study relied on self-report measures, which might be sensitive to biases such as social desirability. Although anonymity was ensured, it might potentially affect the reliability of responses regarding sensitive behaviors like self-injury or suicidal thoughts as self-report tools are inherently limited in capturing all dimensions of mental health and behavioral concerns.

As this study is part of a larger school-based research project, the selection of variables was constrained by the measures included in the broader EPISAM-School dataset. While the variables were theoretically aligned with components of the IMV model, we acknowledge that other relevant constructs—such as defeat, entrapment, or coping strategies—were not available for analysis.

Furthermore, the use of single-item indicators for key constructs such as non-suicidal self-injury and suicide attempt likelihood, although derived from validated instruments and chosen for their clarity and feasibility in large-scale school screenings, may not capture the full dimensionality of the constructs and could compromise reliability. Future studies should consider using full subscales or multi-item measures where possible.

Future studies would benefit from examining how these risk factors evolve throughout different developmental stages of adolescence and incorporate social determinants as highlighted by recent calls for humanized care. Of particular interest would be investigating whether the patterns observed in our 12–16 age group remain stable or change as adolescents grow older. Additionally, research exploring how cultural and socioeconomic factors interact with these risk patterns could provide valuable insights for developing more targeted prevention strategies. Understanding how school climate and peer relationships moderate these risk factors could also offer important directions for school-based interventions.

## Conclusion

This study provides robust evidence supporting a comprehensive model of adolescent suicidal behavior that accounts for critical psychological and environmental factors, reinforcing the effectiveness of a comprehensive model that incorporates both motivational and volitional factors. Emphasizing emotional symptoms, NSSI, and emotional abuse as key elements, our findings align with prior research while highlighting distinct pathways that elevate risk for adolescents. This model's fitness confirms that such risk factors play a crucial role across varied adolescent demographics, affirming the need for holistic approaches in preventive strategies.

By suggesting that these relationships are maintained regardless of gender, provides preliminary evidence that may help inform the design of prevention programs aimed at reducing suicidal ideation and attempts in this vulnerable population.. As we continue to understand the dynamics of suicidal behavior in adolescence, future longitudinal studies are essential to establishing causality and tracking the progression of risk factors. This approach will further enhance the precision and impact of interventions aimed at reducing adolescent suicide rates.

## Supplementary Information

Below is the link to the electronic supplementary material.

Supplementary file1 (DOCX 26 KB)

## Data Availability

No datasets were generated or analysed during the current study.
