# Supplementary material for: Understanding suicidal behavior and gender-specific pathways in an adolescent community sample: a structural model approach
Source: Eur Child Adolesc Psychiatry. 2025 Oct 30;35(3):923–34. doi: 10.1007/s00787-025-02877-5 (PMC13212380; doi:10.1007/s00787-025-02877-5)
Supplement: Supplementary file 1 — Supplementary file1 (DOCX 26 KB) [file 787_2025_2877_MOESM1_ESM.docx]

**SUPPLEMENTARY MATERIAL**

**Table S1. MIMIC model parameters**

| **Index** | **Standard Estimate** |
| --- | --- |
| Test Statistic | 11849.272 |
| Degrees of Freedom | 1148 |
| P-value (Chi-square) | .000 |
| Root Mean Square Error of Approximation (RMSEA) | .078 |
| Comparative Fit Index (CFI) | .965 |
| Tucker-Lewis Index (TLI) | .967 |
| Standardized Root Mean Square Residual | .061 |

**Table S2.** **Regression. General Model.**

| **Variable** | **Estimate** | **Std.Err** | | **Z-value** | | **P value** | | **Std.lv** | | **Std.all** | |  |
| --- | --- | --- | --- | --- | --- | --- | --- | --- | --- | --- | --- | --- |
| Likelyhood of suicide |  | |  | |  | |  | |  | |  | |
| Non Suicidal Self Injury | 1.081 | | .064 | | 16.801 | | .000** | | 1.081 | | .454 | |
| Impulsivity Issues | .081 | | .047 | | 1.720 | | .085 | | .060 | | .053 | |
| Suicidal Ideation | .738 | | .064 | | 11.519 | | .000** | | .574 | | .509 | |
| Age | .017 | | .022 | | .773 | | .439 | | .017 | | .020 | |
| Living with both parents | .120 | | .201 | | .596 | | .551 | | .120 | | .014 | |
| Emotional Abuse | .152 | | .057 | | 2.644 | | .008* | | .124 | | .110 | |
| Suicidal Ideation |  | |  | |  | |  | |  | |  | |
| Emotional Symptoms | .624 | | .097 | | 6.431 | | .000** | | .624 | | .624 | |
| Perceived social support | .013 | | .034 | | .383 | | .701 | | .013 | | .013 | |
| Lack of emotional clarity | .002 | | .078 | | .027 | | .979 | | .002 | | .002 | |
| Concern about errors | .026 | | .035 | | .750 | | .454 | | .027 | | .027 | |
| Age | .059 | | .021 | | 2.846 | | .004* | | .076 | | .097 | |
| Living with both parents | .423 | | .196 | | 2.160 | | .031 | | .544 | | .072 | |
| Emotional Abuse | .182 | | .054 | | 3.349 | | .001* | | .192 | | .192 | |

*Note: *p*<.05; **p<.001

**Table S3.** **Comparison between regressions for Group 1 (Males) and 2 (Females)**

| **Variable** | **P-Value** | | | **Odds Ratio** | | **LB** | | **UB** | |
| --- | --- | --- | --- | --- | --- | --- | --- | --- | --- |
| Likelyhood of suicide~ | | G1 | G2 | G1 | G2 | G1 | G2 | G1 | G2 |
| Non Suicidal Self Injury | | .000** | .000** | 2.70 | 3.019 | 2.20 | 2.561 | 3.32 | 3.560 |
| Impulsivity Issues | | .366 | .090 | 1.07 | 1.106 | .92 | .984 | 1.24 | 1.244 |
| Suicidal Ideation | | .000** | .000** | 1.91 | 2.171 | 1.56 | 1.852 | 2.35 | 2.544 |
| Age | | .057 | .697 | 1.07 | .988 | 1.00 | .932 | 1.14 | 1.048 |
| Living with both parents | | .436 | .778 | 1.24 | 1.095 | .72 | .580 | 2.11 | 2.067 |
| Emotional Abuse | | .005* | .260 | 1.38 | 1.077 | 1.10 | .946 | 1.73 | 1.226 |
| Suicidal Ideation ~ | |  |  |  |  | 1.00 |  |  |  |
| Emotional Symptoms | | .001* | .000** | 1.79 | 1.904 | 1.27 | 1.520 | 2.52 | 2.385 |
| Perceived social support | | .269 | .620 | 1.06 | .979 | .95 | .900 | 1.18 | 1.065 |
| Lack of emotional clarity | | .550 | .372 | 1.09 | .919 | .83 | .765 | 1.44 | 1.105 |
| Concern about errors | | .767 | .203 | .98 | 1.057 | .87 | .969 | 1.11 | 1.152 |
| Age | | .059 | .056 | 1.06 | 1.054 | 1.00 | .998 | 1.13 | 1.114 |
| Living with both parents | | .101 | .133 | 1.46 | 1.716 | .93 | .847 | 2.30 | 3.475 |
| Emotional Abuse | | .006* | .032* | 1.30 | 1.156 | 1.08 | 1.012 | 1.56 | 1.321 |

*Note: *p*<.05; **p<.001
